# Spectrum of complications of severe DKA in children in pediatric Intensive Care Unit

**DOI:** 10.12669/pjms.341.13875

**Published:** 2018

**Authors:** Qalab Abbas, Saba Arbab, Anwar ul Haque, Khadija Nuzhat Humayun

**Affiliations:** 1 Dr. Qalab Abbas, FCPS. Department of Pediatrics and Child Health, Aga Khan University Hospital, Karachi, Pakistan; 2 Dr. Saba Arbab, FCPS. Department of Pediatrics and Child Health, Aga Khan University Hospital, Karachi, Pakistan; 3 Dr. Anwar ul Haque, MD. Department of Pediatrics and Child Health, Aga Khan University Hospital, Karachi, Pakistan; 4 Dr. Khadija Nuzhat Humayun, FCPS. Department of Pediatrics and Child Health, Aga Khan University Hospital, Karachi, Pakistan

**Keywords:** Pediatric, Complications, Pediatric intensive care unit, Diabetic ketoacidosis

## Abstract

**Objectives:**

To describe the spectrum of complications of Diabetic Ketoacidosis (DKA) observed in children admitted with severe DKA.

**Methods:**

Retrospective review of the medical records of all children admitted with the diagnosis of severe DKA in Pediatric Intensive Care Unit (PICU) of the Aga Khan University Hospital, from January 2010 to December 2015 was done. Data was collected on a structured proforma and descriptive statistics were applied.

**Results:**

Total 37 children were admitted with complicated DKA (1.9% of total PICU admission with 1.8% in 2010 and 3.4% in 2015). Mean age of study population was 8.1±4.6 years and 70% were females (26/37). Mean Prism III score was 9.4±6, mean GCS on presentation was 11±3.8 and mean lowest pH was 7.00±0.15. Complications observed included hyperchloremia (35.94%), hypokalemia (30.81%), hyponatremia (26.70%), cerebral edema (16.43%), shock (13.35%), acute kidney injury (10.27%), arrhythmias (3.8%), and thrombotic thrombocytopenic purpura (5.4%), while one patient had myocarditis and ARDS each. 13/37 children (35%) needed inotropic support, 11/37 (30%) required mechanical ventilation while only one patient required renal replacement therapy. Two patients (5.4%) died during their PICU stay.

**Conclusion:**

Hyperchloremia and other electrolyte abnormalities, cerebral edema and AKI are the most common complications of severe DKA.

## INTRODUCTION

The incidence of Diabetes Mellitus (DM) is increasing worldwide.^1^ Around 30% of children with Type-I DM present with diabetic ketoacidosis (DKA) at diagnosis and many develop DKA during the course of the disease.^2^ DKA is the leading cause of death in this population most commonly due to cerebral edema.^3^ During an episode of DKA, there are multiple abnormal processes going on in the body including fluid shifts, decreased perfusion, and deranged pH which affects many functions and causes electrolyte abnormalities. All of these can lead to many body systems and organs being affected.^4^

The most commonly affected tissue is brain leading to cerebral edema. Because of its high morbidity and mortality, cerebral edema has been the main focus of research in DKA patient.^5^ But DKA has been described in literature to be associated with many other complications including DKA associated cerebral injury, electrolyte imbalance, vascular, renal, cardiopulmonary and other complications.^4^ Reports on isolated complications of DKA have been published in literature.^5^-^7^ But there is a dearth of literature on all complications of DKA in children which can lead to greater morbidity and prolonged length of stay.^6^,^8^ We describe our experience of complications of severe DKA observed in our pediatric intensive care unit (PICU).

## METHODS

Retrospective review of medical records of all the children (age one month – 16 years) admitted to PICU of Aga Khan University Hospital (AKUH) with diagnosis of severe DKA from January 2009 to December 2015 was done after approval from ethical review committee of hospital (4287-Ped-ERC-16). Total 37 children were admitted in PICU with severe DKA during the study period and all were included in the study. Data was collected on a structured proforma and included demographic details, admitting diagnosis, pertinent clinical variables, laboratory and imaging studies as well as the therapeutic interventions done in PICU. AKUH is 560 bedded tertiary care facilities with four bedded PICU. DKA was diagnosed based on biochemical criteria including blood glucose of >200mg/dl, venous pH<7.3 or bicarbonate <15 mmol/L and ketonemia or ketonuria.^9^ Severity of DKA was categorized based on acidosis as follows.^9^

### Mild DKA

venous pH between 7.21 – 7.30 or bicarbonate between 10 mmol/L - 15 mmol/L.

### Moderate DKA

venous pH between 7.11 – 7.20 or bicarbonate between 5 mmol/L - 10 mmol/L.

### Severe DKA

venous pH anywhere < 7.10 or bicarbonate <5 mmol/L.

Only severe and or complicated DKA patients were admitted in PICU while all mild to moderate DKA were managed in special care unit under care of a pediatric endocrinologist. DKA was managed as per International Society for Pediatric and Adolescent Diabetes (ISPAD) guidelines using two bag systems.^9^,^10^ New onset diabetes was defined as patients who had presented for the first time and were undiagnosed previously. Cerebral edema was diagnosed when there was low Glasgow coma scale (GCS) (<13) accompanied by one or more other signs of raised intracranial pressure (hypertension and bradycardia with or without breathing pattern abnormalities, pupillary abnormalities, squint, blurred disk margin, decerebrate or decorticate posturing, respiratory arrest or radiological evidence of moderate to severe edema on computed tomography).^5^ Stroke was diagnosed if there was evidence of focal neurological deficit and radiological evidence of cerebral infarction.^5^ Shock was defined as tachycardia with capillary refill time > 3 seconds and cold peripheries with or without hypotension as per pediatric advanced life support (PALS) guidelines.^11^ Myocarditis was diagnosed based on clinical signs of shock (tachycardia, poor capillary refill, and hypotension) along with raised Troponin I and low voltage ECG and low ejection fraction on echocardiography. Acute kidney injury was diagnosed by modified pediatric Risk, Injury, Failure, Loss, End stage renal disease (pRIFLE) criteria.^12^ Data was entered and analyzed using Statistical software for social sciences (SPSS) version 20. Results are presented as mean with standard deviation and frequency with percentages. Chi square test was used to determine the risk of developing complications between different groups and p value of <0.05 was taken as significant.

## RESULTS

Total 37 children were admitted in PICU with severe DKA during the study period. Complications were observed in 17 patients. There was an increase in number of DKA admissions to PICU from 1.8% of total PICU admissions in 2010 to 3.4% in 2015. Mean age was 8.1 ± 4.6 years; youngest was 10 months of age and 70% (26/37) were females. Mean Pediatric Risk of mortality score version III (PRISM III) was 9.4 ± 6. Mean GCS on presentation was 11 ± 3.8 with 16 (43%) having GCS <13 (Table-I).

**Table-I:** Demographic characteristics of study population (n=37).

Variable	Frequency	Percentage
Age (Mean ± SD*) in years	8.1 ± 4.6	
Weight (Mean ± SD*)	25.22 ± 13.76	
Gender (Female)	26	70
GCS (Mean ± SD*)	11 ± 3.8	
Complications	20	54%
PRISM III Score (Mean ± SD*)	9.4 ± 6	
Newly Diagnosed	28	75
Family History	
Type-I	3	8
Type-II	11	29
Outcome (Survived)	35	95

* SD: Standard Deviation

Thirteen patients had shock (circulatory) on presentation and one patient was later on diagnosed as myocarditis. Four patients had cerebral edema, one patient developed ischemic stroke and arrhythmias were observed in three patients (all had ventricular tachycardia). Acute kidney injury developed in 10 (27%) patients out of which only one patient required renal replacement therapy while one patient developed acute respiratory distress syndrome. Other details are shown in Table-II. Mean lowest pH was 7.00 ± 0.15. Mean time to resolution of DKA was 23 hours.

**Table-II:** Spectrum of complications of DKA in children admitted in PICU (n=37).

Complication		Frequency	Percentage
Cardiac	Shock requiring Inotropic support	13	35
	Arrhythmias	3	8
	Myocarditis	1	2.7
CNS	Cerebral edema	16	43
	Stroke	1	2.7
Renal	AKI	10	27
	Hyperchloremia	35	94
	Hypokalemia	30	81
	Hyponatremia	26	70
	Hypernatremia and hyperkalemia	17	46
Respiratory (ARDS)		1	2.7
Hematological (TTP)		2	5.4
Sepsis (Positive Blood culture)		5	13.5

**Table-III:** Values of different laboratory data in Patients with Severe DKA.

Variable	Mean	Std. Deviation
Highest Glucose (g/dl)	579.24	220.757
Lowest pH	7.0022	.14976
Highest Base Deficit	-23.203	-5.8725
Lowest Potassium (mmol/L)	2.524	.6563
Highest Potassium (mmol/L)	4.997	1.2289
Lowest Sodium (mmol/L)	131.73	6.389
Highest Sodium (mmol/L)	145.62	9.266
Highest Chloride (mmol/L)	120.86	9.364
Lowest Phosphorus (mg/dl)	3.035	1.6056

Inotropic support in PICU was required in 13/37 (35%) of the children and 11/37 (30%) required mechanical ventilation while one patient required renal replacement therapy. Plasma pheresis was done in two patients for TTP. Two of the patients died during their PICU stay, one developed arrhythmia (ventricular tachycardia) refractory to treatment and other presented in MODS and expired after one day. Mean length of PICU stay was 2 ± 2.8 days. There was no association of gender, age with development of any specific complications. (p=0.94 and p=0.96 respectively). Complications were more common in newly diagnosed patients and in patients above eight years but they were not statistically significant (p>0.05) (Table IV, V).

**Table-IV:** Association of Age with complications.

Complications	Age <8 years	Age > 8 years	P value	OR, 95%CI
Cerebral edema	0	4	0.08	1.22, 1.00 – 1.48
Stroke	0	1	0.40	1.04, 0.95 – 1.14
Arrhythmias	0	3	0.13	1.15, 0.98 – 1.36
Shock	6	7	0.60	0.70, 0.17 – 2.75

OR = odds ratio, 95% CI= 95% confidence interval

**Table-V:** Association of type of diabetes with complications.

Complications	Newly Diagnosed Diabetes	Known Diabetic	P value	OR, 95%CI
Cerebral edema	1	3	0.97	0.96, 0.087–10.57
Stroke	1	0	0.56	1.03, 0.96–1.14
Arrhythmias	1	2	0.07	0.13, 0.01–1.64
Shock	12	1	0.08	6.0, 0.65–54.66

OR = odds ratio,95% CI= 95% confidence interval

## DISCUSSION

As the incidence of Type-I diabetes mellitus is increasing, the incidence of DKA is also increasing and more patients are being admitted in PICU because of DKA.^2^,^8^,^13^ Same is shown in our study that the number of admissions in PICU due to severe DKA has almost doubled from 1.8% in 2010 to 3.6% in 2015. Previously only cerebral edema and few electrolyte abnormalities have been described as the complications of DKA and its treatment. But with the increasing incidence more and more complications are seen and described some of which are rare.^4^,^6^ Females comprise two third of these patients which is in contrast to male predominance shown by Jayashree et al.^8^ This could be due to hormonal influences leading to more brittle nature of disease in females as compared to males and also less preference to their care. As shown in many earlier studies 75% of our patients were newly diagnosed but in contrast western data showed it to be 25%.^2^,^8^ Overall rate of complications observed in our study is 54% which is high as compared to what is already reported.^6^,^8^ But only two of our patients died in contrast to earlier reports which show it to be 13.2%.^8^ Those two died of causes probably unrelated to DKA. The most common complications observed were electrolytes abnormalities including Hyperchloremia (35.94%), hypokalemia (30.81%), and hyponatremia (26.70%) followed by cerebral edema (16.43%) and shock requiring inotropic support (13.35%). All of these complications are higher than what are reported already by Jayashree and Afshin et al.^6^,^8^ This could be related to the severity of DKA, as well aggressive therapy and also late presentation. We found a very high incidence of hyperchloremia in our study and this has been found to be associated with delayed recovery from DKA.^14^ Although up till now the recommended intravenous fluid therapy for DKA is isotonic fluids and this therapy can lead to hyperchloremia but may be in near future more customized fluids (like balanced salt solutions) will be increasingly used to prevent hyperchloremia and its adverse effects.^15^ Mean length of PICU stay was two days which is again less than that earlier reported from PICU.^8^ Other less common complications observed included thrombotic thrombocytopenic purpura (TTP) in two patients which were managed with plasmaphresis and other supportive therapies. This has been described earlier by Khan et al.^7^ Shock requiring inotropic support was observed in 13 patients and one patients developed stroke and AKI was diagnosed in 10 patient (27 %) out of which only one required renal replacement therapy and all of them recovered.

### Strength and Limitation

This is first comprehensive study from Pakistan describing the whole spectrum of complications of DKA in children. Our study has the limitation of being a single center retrospective study with small sample size.

## CONCLUSIONS

Hyperchloremia and other electrolyte abnormalities, cerebral edema and AKI are the most common complications of severe DKA. Despite these complications the outcome in severe DKA patients is very good.

### Authors Contribution

**QA and AH** conceived, designed and did statistical analysis, writing & editing of manuscript.

**SA and QA** did data collection and manuscript writing.

**QA, SA, AH and KNH** did review and final approval of manuscript.
